# ELF3 promotes hyperglycemia-induced corneal epithelial senescence and IL-2 attenuates ELF3-associated signaling

**DOI:** 10.3389/fmolb.2026.1812782

**Published:** 2026-06-24

**Authors:** Sujun Zhou, Hong Zhang

**Affiliations:** Key Laboratory of Basic and Clinical Research of Heilongjiang Province, Eye Hospital, The First Affiliated Hospital of Harbin Medical University, Harbin, Heilongjiang, China

**Keywords:** cellular senescence, diabetic keratopathy, ELF3, IL-2, NF-κB pathway

## Abstract

**Introduction:**

Diabetic keratopathy constitutes a prevalent ocular complication of diabetes mellitus, clinically manifested as delayed corneal epithelial repair, recurrent epithelial defects, and potential corneal ulceration. Currently, the underlying molecular mechanisms and effective therapeutic strategies remain incompletely defined. This study integrates transcriptomic analysis with computational pharmacology and experimental validation. The results suggest that ELF3 is associated with corneal epithelial senescence in diabetes and identify IL-2 as a potential modulator of ELF3-associated signaling.

**Methods:**

Transcriptomic analysis of Gene Expression Omnibus (GEO) datasets, combined with transcription factor enrichment, was performed to identify principal transcriptional regulators. In human corneal epithelial cells (HCECs) stimulated with high glucose, EdU assays, wound-healing assays, senescence-associated β-galactosidase (SA-β-gal) staining, and Western blotting were employed to assess HCEC proliferation, migration, and senescence. Following siRNA-mediated ELF3 knockdown or plasmid-driven overexpression, phenotypic and protein-level changes were evaluated. Transcriptome-guided drug prediction and molecular docking were performed to identify candidate ELF3 modulators. Pathways associated with ELF3 were screened *via* RNA sequencing and subsequently validated by Western blotting. A type 1 diabetic mouse model was established using streptozotocin (STZ). The therapeutic efficacy of IL-2 on corneal senescence in diabetic mice was assessed using immunohistochemistry, SA-β-gal staining, and sodium fluorescein staining.

**Results:**

Transcriptomic analysis identified ELF3 as a critical transcription factor associated with diabetic keratopathy. In HCECs under high-glucose conditions, ELF3 expression was significantly upregulated, coinciding with reduced proliferation, impaired migration, and accelerated senescence. ELF3 knockdown attenuated these impairments, whereas its overexpression exacerbated them. Drug prediction and molecular docking identified IL-2 as a potential modulator of ELF3-associated signaling. RNA sequencing analysis indicated that the NF-κB pathway is associated with ELF3-mediated regulation of cellular senescence. *In vitro*, IL-2 downregulated ELF3 expression, suppressing the NF-κB pathway and attenuating cellular senescence. In diabetic mice, topical IL-2 application reduced ELF3 levels, decreased senescence markers, and accelerated corneal epithelial wound healing.

**Conclusion:**

These findings establish ELF3 as a critical driver of hyperglycemia-induced corneal epithelial senescence. IL-2 attenuates senescence and promotes corneal wound healing while suppressing ELF3-associated signaling and NF-κB pathway activation.

## 1 Introduction

The homeostasis of the corneal epithelium relies on continuous epithelial cell proliferation, migration, and differentiation. Diabetes mellitus and persistent hyperglycemia disrupt these essential physiological processes, impair corneal epithelial barrier integrity, and ultimately drive the development of diabetic keratopathy (Rosenberg et al., 2000; Xu and Yu, 2011). However, the molecular mechanisms by which chronic hyperglycemia perturbs corneal epithelial homeostasis remain incompletely understood. Clinically, diabetic corneal involvement is prevalent yet frequently overlooked, presenting as decreased corneal sensation, delayed epithelial wound healing, and recurrent epithelial erosion (Han et al., 2019; Weng et al., 2022). Conventional therapies show limited efficacy against diabetic keratopathy. Common approaches such as artificial tears, antibiotic eye ointments and bandage contact lenses merely alleviate symptoms temporarily, rather than reverse the underlying pathological process (Priyadarsini et al., 2020; Ladea et al., 2024). To address these clinical challenges, it is essential to elucidate the molecular mechanisms of diabetic keratopathy and identify novel therapeutic targets.

Cellular senescence, a state of permanent cell-cycle arrest, is understood to be a key driver of chronic diabetic complications. Substantial evidence indicates that a persistent high-glucose environment promotes cellular senescence in various tissues and cell types (Palmer et al., 2021; Liu et al., 2023; Zhang et al., 2023). In the corneal epithelium, persistent hyperglycemia drives the accumulation of reactive oxygen species (ROS), the secretion of pro-inflammatory cytokines, and mitochondrial dysfunction (Zhu et al., 2019; Mussi et al., 2022; Buonfiglio et al., 2024). These stress factors act synergistically on the cell cycle regulatory system, causing G1/S phase arrest and suppressing epithelial cell proliferation and migration, ultimately inducing cellular senescence (Yang et al., 2025). However, the specific regulatory mechanisms underlying cellular senescence—particularly the involved upstream transcriptional regulators and signaling pathways—remain poorly elucidated. Identifying stress-responsive transcriptional regulators involved in diabetic corneal epithelial senescence may therefore provide new mechanistic insight and therapeutic opportunities.

At the transcriptional level, epithelial-specific transcription factors play critical roles in regulating cell fate under environmental stress. ELF3 (E74-like ETS transcription factor 3) is an epithelial-enriched member of the ETS family involved in epithelial differentiation and homeostasis (Sharrocks, 2001). Previous studies have also implicated ELF3 in epithelial stress responses and inflammatory signaling in several epithelial tissues, including the intestine, skin, and kidney (Li et al., 2015; Sakurai et al., 2019; Zhang et al., 2025). However, despite these advances in other organ systems, its function in the corneal epithelium under metabolic stress remains unknown. In particular, whether hyperglycemia drives epithelial senescence through ELF3 has not been directly examined. We therefore hypothesized that ELF3-associated epithelial stress signaling contributes to hyperglycemia-induced corneal epithelial senescence and impaired epithelial repair.

In this study, the key transcriptional drivers of hyperglycemia-induced corneal epithelial senescence were identified, and a potential therapeutic agent was explored. Through integrative transcriptomic analysis and functional validation, the transcription factor ELF3 was identified as a key driver of high-glucose-induced epithelial senescence and impaired wound healing. ELF3 upregulation was associated with NF-κB pathway activation under hyperglycemic conditions. Furthermore, *via* transcriptome-guided drug prediction, IL-2 was identified as a potential modulator of ELF3-associated signaling, and it was demonstrated that topical IL-2 application attenuated senescence and promoted epithelial repair in diabetic mice. Our findings delineate an ELF3-associated senescence axis in diabetic keratopathy and suggest a potential therapeutic direction.

## 2 Materials and methods

### 2.1 Differential gene expression analysis and transcription factor prediction

The Gene Expression Omnibus (GEO) database hosts high-throughput sequencing datasets and serves as a resource for biological research. To identify key transcription factors that drive epithelial stress responses in diabetic corneal epithelial senescence, we integrated two RNA-seq datasets. Given that dry eye-related corneal epithelial stress responses share highly conserved molecular pathways with diabetic epithelial injury (Bohm et al., 2023; Chen et al., 2025), we combined a diabetic mouse corneal dataset (GSE180490) (Zhang et al., 2021) with a corneal epithelial cell dataset from a dry eye stress model (GSE244787) (Kado Abdalkader et al., 2024). This integrative strategy helps to distinguish stress-responsive transcription factors that originate from the corneal epithelium amid the complex signaling milieu of the diabetic cornea. The expression matrices from the datasets were extracted, and systematic data cleaning was performed. The limma-voom workflow was employed to analyze the differentially expressed genes. Transcription factor prediction was performed using ChEA3, which integrates ChIP-seq resources, transcriptome perturbation signatures, and multiple gene-set collections to rank candidate regulators. To improve reliability, only transcription factors enriched in at least two independent evidence libraries were retained, with emphasis placed on candidates supported by ChIP-seq evidence. Based on these analyses, ELF3 was selected for subsequent mechanistic interrogation.

### 2.2 Cell culture and experimental treatments

The SV40-Adeno vector transformed human corneal epithelial cell line (HCEC) was purchased from Wuhan Pricella Biotechnology Co., Ltd. Cell culture was performed with DMEM supplemented with 10% FBS and 1% penicillin/streptomycin in a humidified incubator maintained at 5% CO_2_ and 37 °C. D-glucose (8.96 g) was dissolved in 50 mL of DMEM to prepare a 1 M D-glucose solution, followed by sterile filtration (0.22 μm). Recombinant human IL-2 (Gibco, 200-02) was dissolved in sterile PBS containing 0.1% BSA to prepare a 50 μg/mL stock solution. Cells were divided into the following groups: (1) normal glucose (NG) with 5.5 mM D-glucose; (2) high-glucose (HG) with 25, 35, 50, and 75 mM D-glucose; (3) osmotic control (mannitol) with 5.5 mM D-glucose and 29.5 mM mannitol; and (4) high glucose supplemented with IL-2 (HG + IL-2) at concentrations of 10, 20, and 40 ng/mL. According to previous studies, a 72 h induction period was adopted to induce cellular senescence (Yu et al., 2025; Zhong et al., 2025).

### 2.3 EdU assay

The EdU assay kit (Beyotime, C0075S) was used to assess cell proliferation. After treatment, the EdU reagent was diluted with culture medium at a 1:500 ratio to prepare the working solution. HCECs were fixed with fixative, washed with PBS, and then incubated with the reaction solution. Cell nuclei were stained and labeled with DAPI. The samples were examined *via* a fluorescence microscope.

### 2.4 Wound healing assay

Prior to seeding, parallel guide lines were marked on the bottom of the six-well plates to facilitate imaging at consistent positions. After treatment, monolayers were scratched using a 200 μL pipette tip. Detached HCECs were eliminated with gentle PBS washes. HCECs were then maintained in medium containing 2% serum. Images were acquired at 0 h and 24 h.

### 2.5 Senescence staining

The SA-β-gal Staining Kit (Beyotime, C0602) was used to determine cell senescence in both cultured cells and whole-mount corneas. Samples were gently fixed for 15 min. After three PBS washes, samples were incubated with SA-β-gal staining solution in a CO_2_-free 37 °C incubator overnight. Blue-stained senescent cells or areas were examined under a light microscope.

### 2.6 Western blotting

The HCECs in six-well plates were washed twice with PBS and lysed with RIPA buffer. Lysates were disrupted by sonication (three cycles, every 5 min), followed by centrifugation. For nuclear protein extraction, a Nuclear and Cytoplasmic Protein Extraction Kit (Beyotime, P0027) was employed to extract nuclear proteins following the manual. The protein samples were mixed with 5× loading buffer and denatured at 100 °C for 10 min. After SDS-PAGE separation, the protein samples were transferred to a methanol-activated PVDF membrane. Following blocking with 5% skim milk powder, the PVDF membrane was incubated overnight at 4 °C with primary antibody solutions: anti-p16 (Proteintech, 10883-1-AP, 1:1500), anti-p21 (Abcam, ab109520, 1:2,500), anti-ELF3 (Abclonal, A5236, 1:2500), anti-phospho-p65 (Proteintech, 82335-1-RR, 1:1500), anti-IκBα (Proteintech, 10268-1-AP, 1:1500), anti-NF-κB p65 (Proteintech, 80979-1-RR, 1:1000), and anti-β-actin (Proteintech, 60008-1-Ig, 1:5000). The membrane was washed with PBST and then incubated with the secondary antibody (1:5000) at room temperature for 1 h (Beyotime, A0208). Signals were developed using ECL reagents (A:B = 1:1) and imaged with a gel documentation system.

### 2.7 Transfection of HCECs with siRNA

Three independent siRNAs targeting different regions of the ELF3 transcript and a negative control siRNA were commercially designed and synthesized by Tsingke Biotechnology Co., Ltd. (Beijing, China). HCECs were transfected at 60%–80% confluence. siRNA and negative control siRNA (NC) were dissolved in DEPC-treated water and prepared as 10 μM stocks. For each well of a six-well plate containing 2 mL of complete medium, transfection complexes were prepared by diluting 20 μL of siRNA solution in 200 μL of transfection buffer and subsequently adding 4 μL of transfection reagent (Polyplus, 101000046). The mixture was vortexed for 10 s and briefly spun down. Complexes were added evenly to the medium, which was replaced 6 h post-transfection. Cells were harvested 48 h later for subsequent assays.

### 2.8 Plasmid transfection for ELF3 overexpression

The pcDNA3.1-ELF3 plasmid was obtained from Tsingke Biotech Co., Ltd. For plasmid transfection, plasmid DNA was diluted in transfection buffer and mixed with Lip8000 reagent (Beyotime, C0533). After incubation at room temperature for 15 min, the complexes were added to the medium, which was replaced 6 h post-transfection. Cells were harvested 48 h later for subsequent functional assays.

### 2.9 Drug prediction analysis

A reverse pharmacology strategy was employed to identify candidate compounds that may modulate ELF3 expression or activity based on transcriptomic correlation patterns. The derivation of an ELF3 co-expression signature was based on GTEx RNA-seq data from ocular tissues, leveraging a large sample size and consistent tissue source to improve correlation stability (Consortium, 2013). Pearson correlation coefficients were computed between ELF3 and all genes genome-wide; significantly co-expressed genes were utilized as a transcriptional signature of ELF3-associated programs. This signature was incorporated into a gene set enrichment analysis (GSEA) framework and compared against drug-perturbation expression signatures from DrugBank. In GSEA outputs, a positive normalized enrichment score (NES >0) indicated that the drug-induced expression pattern was consistent with the ELF3 positively correlated gene set, suggesting potential enhancement of ELF3-associated programs. Conversely, a negative score (NES <0) indicated an opposite pattern and potential suppression of ELF3 expression or function.

### 2.10 Molecular docking

To predict potential interactions between ELF3 and IL-2, a structure-based molecular docking strategy was employed. First, the three-dimensional protein structures of ELF3 and IL-2 were modeled separately using AlphaFold3, and the models were subjected to quality evaluation. Subsequently, protein–protein docking was performed using the ZDOCK program to generate multiple sets of candidate complex conformations. Docking poses were ranked by docking scores, and the top-scoring model was selected for visualization and analysis in PyMOL to examine potential binding sites and key interacting residues.

### 2.11 RNA extraction and library construction

RNA from HCECs was extracted with total RNA isolation reagent (Biosharp, BS258A) according to the TRIzol reagent protocol. Samples with an RNA integrity score above 7 were utilized, and integrity was further validated *via* agarose gel electrophoresis. cDNA fragments in the library were clustered around 300 bp in length. The HCEC cDNA library was sequenced using the Illumina Novaseq™ 6000 platform (LC-Bio Technologies Co., Ltd., Hangzhou, China) in strict accordance with the manufacturer’s protocol. A paired-end 150 bp (PE150) sequencing strategy was employed.

### 2.12 Bioinformatics analysis of RNA-seq

Raw sequencing data for HCECs were preprocessed using Fastp software (https://github.com/OpenGene/fastp). Adapter sequences were removed. Paired-end reads from each sample were assembled into transcripts by StringTie (https://ccb.jhu.edu/software/stringtie) with default parameter settings. Transcriptome data were integrated with gffcompare (https://github.com/gpertea/gffcompare/) to construct a comprehensive, non-redundant transcriptome assembly. Transcript expression levels were estimated and quantified across all samples *via* StringTie. The edgeR package in R was employed to identify differentially expressed mRNAs. A parametric F-test was applied with a significance threshold of *p* < 0.05. Bioinformatic analysis was performed using the OmicStudio tools at https://www.omicstudio.cn/tool.

### 2.13 Animal model

An STZ-induced type 1 diabetes model was established in C57BL/6 mice, which were housed in a facility that was specific pathogen free (SPF). All procedures were approved by the Laboratory Animal Welfare and Ethics Committee of Zunyi medical university (ZMU21-2,512-008) and strictly adhered to the Association for Research in Vision and Ophthalmology Statement for the Use of Animals in Ophthalmic and Vision Research. Citrate buffer was formulated by mixing citric acid and sodium citrate solutions at a ratio of 1:1.32 and sterile-filtered (0.22 μm). STZ was freshly prepared in citrate buffer on each injection day and administered intraperitoneally at 50 mg/kg for five consecutive days. At 16 weeks post-injection, mice with blood glucose ≥ 300 mg/dL were selected for further experimental procedures (Chen and Hu, 2021). For all *in vivo* experiments described in this study, three mice were included in each experimental group.

### 2.14 Animal topical IL-2 treatments

For topical treatment, recombinant human IL-2 (Gibco, 200-02) was diluted in sterile PBS with 0.1% BSA to 300 ng/mL. IL-2 was applied to the conjunctival sac at 5 μL per eye, three times daily for three consecutive days. Control mice received vehicle (PBS +0.1% BSA). All mice were euthanized on day 4 for corneal collection.

### 2.15 Corneal epithelial wound healing assay

Mice were anesthetized with tribromoethanol. A 2-mm trephine was used to demarcate the central cornea, and the epithelium was removed using a debridement tool (Algerbrush II) under a microscope to avoid stromal injury. Fluorescein staining was performed immediately after injury (0 h) and repeated at 12, 24, and 48 h. Images were acquired using a slit-lamp microscope with cobalt-blue illumination. Epithelial defect areas and wound healing rates were quantified with ImageJ.

### 2.16 Immunocytochemistry

Enucleated eyeballs were fixed, dehydrated, embedded in paraffin, and sectioned. After deparaffinization and antigen retrieval (sodium citrate–EDTA; high-pressure, 3 min), sections were permeabilized, and endogenous peroxidase activity was blocked. Samples were incubated overnight at 4 °C with primary antibodies against ELF3 (Invitrogen, PA5-120996, 1:100), p16 (CST, 23200T, 1:100), and p21 (Abcam, ab188224, 1:1000). Subsequently, samples were incubated with the secondary antibody (Beyotime, A0208, 1:50) solution at 37 °C for 1 h. Signals were developed with DAB. Samples were then counterstained with hematoxylin, dehydrated, cleared, mounted, and examined under a light microscope.

### 2.17 Statistical analysis

Experiments were performed independently three times. Statistical analyses were conducted *via* GraphPad Prism 10 (GraphPad, San Diego, CA, United States). Quantitative data are presented as mean ± standard deviation (SD). For comparisons between two groups, data were analyzed using an unpaired Student’s t-test. For multiple-group comparisons, one-way analysis of variance (ANOVA) was applied, followed by Dunnett’s multiple comparisons test for *post hoc* analysis. *p* < 0.05 indicated statistical significance.

## 3 Results

### 3.1 Integrative transcriptomic analysis identifies ELF3 as a key transcriptional regulator

**FIGURE 1:**
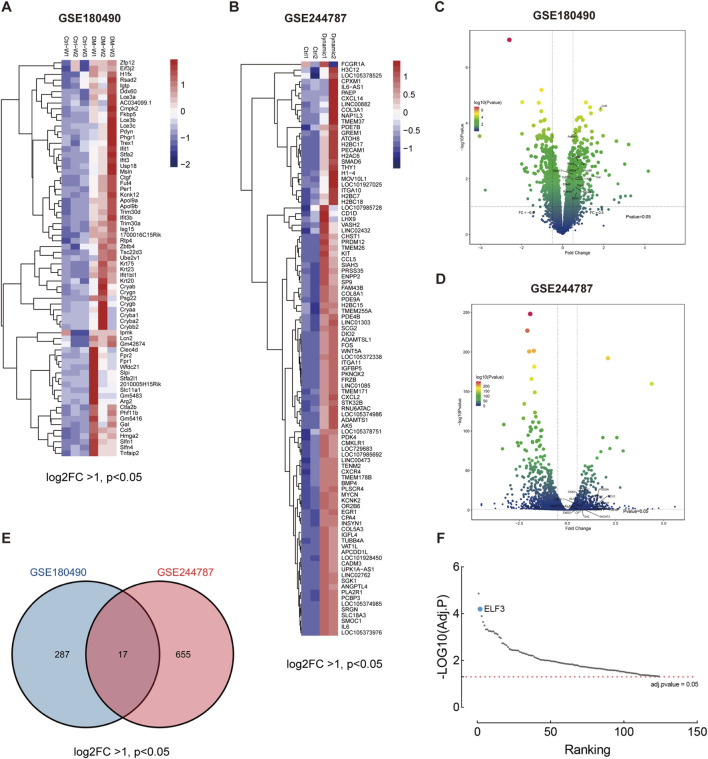
Bioinformatics identification of the transcription factor ELF3 using datasets GSE180490 and GSE244787 **(A,B)** Cluster heatmap analysis of differentially expressed genes from Gene Expression Omnibus (GEO) datasets GSE180490 and GSE244787 **(C,D)** Volcano plots of differentially expressed genes from datasets GSE180490 and GSE244787 **(E)** Venn diagram illustrating upregulated gene intersections between datasets GSE180490 and GSE244787 **(F)** Transcription factor enrichment analysis of upregulated genes *via* the ChEA3 (ChIP-Enrichment Analysis 3) platform.

To investigate the transcriptional changes underlying diabetic epithelial stress responses, differential expression analyses were performed using publicly available transcriptomic datasets, GSE180490 and GSE244787. Hierarchical clustering heatmaps of the GSE180490 and GSE244787 datasets revealed significant differences in gene expression profiles between the control and treatment groups, suggesting that both diabetic status and desiccation stress induce characteristic transcriptomic changes in the corneal epithelium (Figures 1A,B). Corresponding volcano plots (Figures 1C,D) visually presented the distribution of differential genes. Both datasets exhibited substantial numbers of upregulated genes under pathological conditions. Venn diagram analysis identified 287 upregulated genes in GSE180490 and 655 upregulated genes in GSE244787, with 17 genes commonly upregulated in both datasets (Figure 1E). Transcription factor enrichment analysis showed that ELF3 ranked second among all enriched transcription factors (Figure 1F). It was selected as the candidate for subsequent functional validation based on its epithelial specificity and strong biological relevance to corneal epithelial dysfunction.

### 3.2 High glucose suppresses HCEC proliferation and migration while promoting cellular senescence and ELF3 upregulation

**FIGURE 2:**
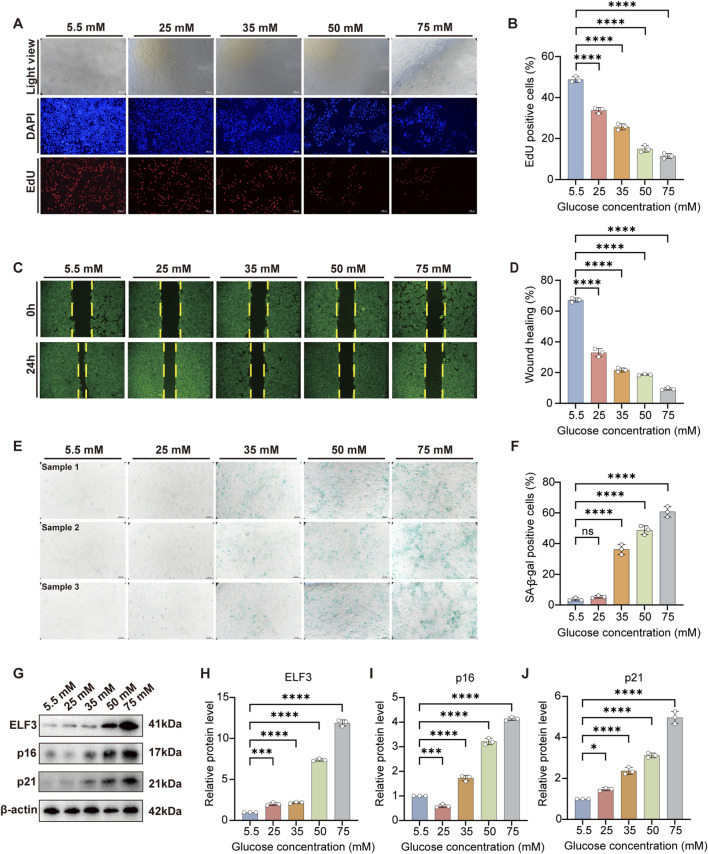
Significant upregulation of ELF3 in human corneal epithelial cells (HCECs) under high-glucose conditions **(A,B)** EdU staining and statistical analysis assessing the effect of different glucose concentrations on HCEC proliferation. Scale bar, 100 μm **(C,D)** Scratch assays and statistical analysis evaluating the effect of different glucose concentrations on HCEC migration. Scale bar, 100 μm **(E,F)** SA-β-gal staining and statistical analysis of HCECs exposed to different glucose concentrations. Scale bar, 50 μm **(G–J)** Western blotting and statistical analysis of ELF3, p16, and p21 protein expression in HCECs treated with different glucose concentrations. Data are presented as mean ± S.D.; n = 3, *p < 0.05, **p < 0.01, ***p < 0.001, ****p < 0.0001; ns: not significant.

HCECs were treated with glucose to establish an *in vitro* high-glucose model. EdU staining to assess cell proliferation demonstrated that as glucose concentration increased, the proportion of EdU-stained corneal epithelial cells gradually decreased, reflecting weakened cell proliferation activity (Figures 2A,B). Scratch assays performed to evaluate cell migration demonstrated that as glucose concentration increased, the corneal epithelial cell wound-healing area decreased, with migration ability weakening in a dose-dependent manner (Figures 2C,D). Senescence staining revealed that a high-glucose concentration of 35 mM induced significant cellular senescence in HCECs (Figures 2E,F). This finding was confirmed by Western blotting, which showed significantly upregulated expression of p16 and p21 beginning at this concentration (Figures 2G,I,J). Consequently, this concentration was selected for subsequent interventions. Western blotting also demonstrated that ELF3 protein levels were upregulated as the glucose concentration increased (Figures 2G,H). To exclude the influence of osmotic pressure, HCECs were treated with mannitol at an osmolarity equal to the high-glucose treatment. Senescence staining in the mannitol group showed no significant increase in SA-β-gal activity relative to the NG group (Supplementary Figures S1A, B). Western blotting further indicated that the expression of ELF3, p16, and p21 was markedly increased only in the HG group (Supplementary Figures S1C–F). These results demonstrate that under high-glucose stimulation, HCEC proliferation and migration abilities decrease, the senescent phenotype is enhanced, and ELF3 expression is significantly upregulated.

### 3.3 ELF3 regulates HCEC proliferation, migration, and senescence under high-glucose conditions

**FIGURE 3:**
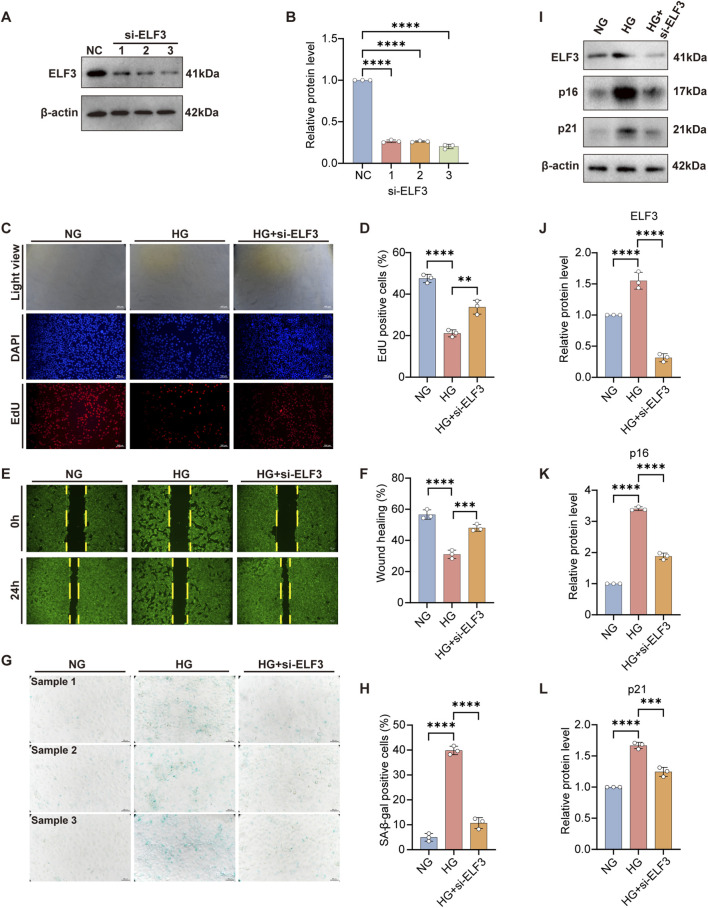
ELF3 knockdown promotes proliferation and migration while suppressing senescence in HCECs under high-glucose conditions **(A,B)** Western blotting and statistical analysis of ELF3 levels in HCECs under high-glucose conditions following ELF3 knockdown **(C,D)** EdU staining and statistical analysis assessing the effect of ELF3 knockdown on HCEC proliferation under high-glucose conditions. Scale bar, 100 μm **(E,F)** Scratch assays and statistical analysis evaluating the effect of ELF3 knockdown on HCEC migration under high-glucose conditions. Scale bar, 100 μm **(G,H)** SA-β-gal staining and statistical analysis of HCECs under high-glucose conditions following ELF3 knockdown. Scale bar, 50 μm **(I–L)** Western blotting and statistical analysis of ELF3, p16, and p21 protein expression in HCECs following ELF3 knockdown. Data are presented as mean ± S.D.; n = 3, *p < 0.05, ‌**p < 0.01, ***p < 0.001, ****p < 0.0001; ns: not significant. Abbreviations: NC: Negative control; NG, normal-glucose-treated HCECs; HG, high-glucose-treated HCECs.

**FIGURE 4:**
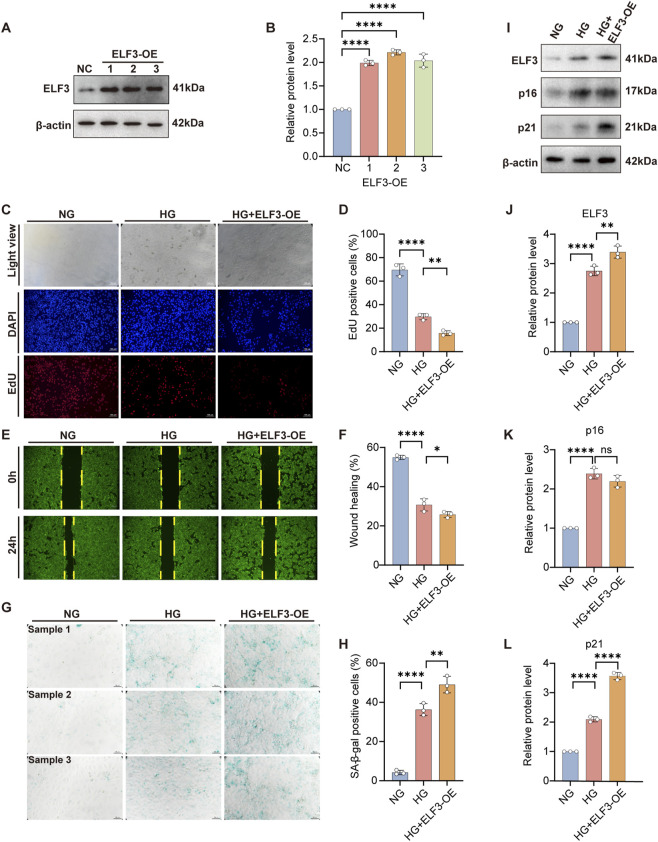
ELF3 overexpression inhibits proliferation and migration while promoting senescence in HCECs under high-glucose conditions **(A,B)** Western blotting and statistical analysis of ELF3 levels in HCECs under high-glucose conditions following ELF3 overexpression **(C,D)** EdU staining and statistical analysis assessing the effect of ELF3 overexpression on HCEC proliferation under high-glucose conditions. Scale bar, 100 μm **(E,F)** Scratch assays and statistical analysis evaluating the effect of ELF3 overexpression on HCEC migration under high-glucose conditions. Scale bar, 100 μm **(G,H)** SA-β-gal staining and statistical analysis of HCECs under high-glucose conditions following ELF3 overexpression. Scale bar, 50 μm **(I–L)** Western blotting and statistical analysis of ELF3, p16, and p21 protein expression in HCECs under high-glucose conditions following ELF3 overexpression. Data are presented as mean ± S.D.; n = 3, *p < 0.05, **p < 0.01, ***p < 0.001, ****p < 0.0001; ns: not significant.

ELF3-knockdown was established in high-glucose HCECs using siRNA (HG + si-ELF3), and a notable reduction in ELF3 protein levels was verified *via* Western blotting (Figures 3A,B). Of the three siRNAs targeting distinct regions of the ELF3 transcript (si-ELF3-1, si-ELF3-2, and si-ELF3-3), si-ELF3-3 was selected for the subsequent functional assays due to its highest knockdown efficiency. To assess the impact of ELF3 knockdown on HCEC function under high-glucose conditions, we performed a series of functional assays. EdU staining showed that the HG + si-ELF3 group exhibited improved proliferation compared with the HG group (Figures 3C,D). Wound-healing assays revealed enhanced migration in the HG + si-ELF3 group (Figures 3E,F). SA-β-gal staining further demonstrated attenuated cellular senescence in si-ELF3-treated cells (Figures 3G,H). Consistent with these phenotypic changes, Western blotting showed that si-ELF3 significantly reduced the expression levels of the senescence-associated proteins p16 and p21 (Figures 3I–L). Conversely, an ELF3 expression plasmid was utilized to establish ELF3-overexpression in high-glucose HCECs (HG + ELF3-OE). Western blotting demonstrated significantly upregulated expression levels of ELF3 (Figures 4A,B). The pcDNA3.1-ELF3-2 plasmid was selected for the subsequent experiments. Functional assays showed that, in contrast to ELF3 knockdown, ELF3 overexpression resulted in significantly impaired proliferation and migration, and enhanced cellular senescence relative to the HG group (Figures 4C–H). Western blotting further confirmed that ELF3-OE significantly increased the expression of p16 and p21 (Figures 4I–L). Collectively, these findings indicate that under high-glucose conditions, ELF3 regulates HCEC proliferation, migration, and senescence.

### 3.4 IL-2 suppresses ELF3-associated signaling and attenuates senescence under high-glucose conditions

**FIGURE 5:**
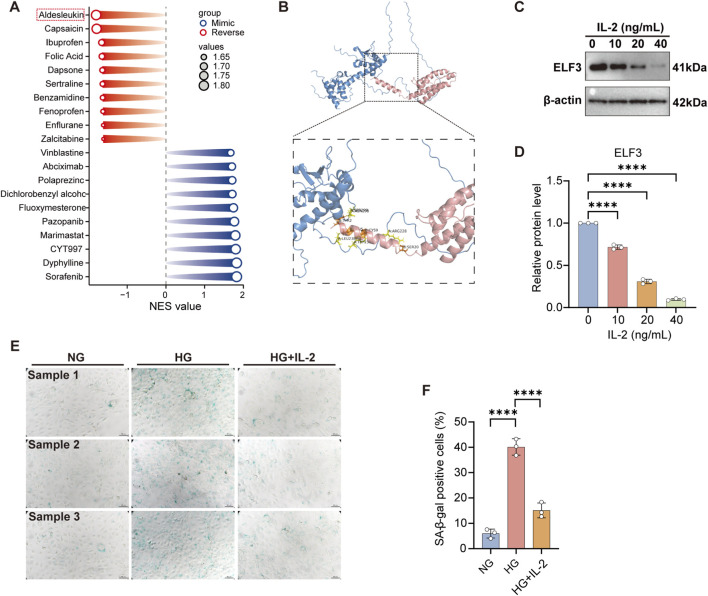
Suppression of cellular senescence in HCECs under high-glucose conditions by IL-2 through modulation of ELF3-associated signaling **(A)** GSEA-based screening identifying potential ELF3 modulators using the DrugBank database. The bar plot illustrates the normalized enrichment score (NES) values of the leading candidates **(B)** Molecular docking analysis suggesting potential structural compatibility between IL-2 and ELF3 **(C,D)** Western blotting and statistical analysis of ELF3 protein expression in HCECs under high-glucose conditions treated with IL-2 **(E,F)** SA-β-gal staining and statistical analysis of HCECs under high-glucose conditions treated with IL-2. Scale bar, 50 μm. Data are presented as mean ± S.D.; n = 3, *p < 0.05, **p < 0.01, ***p < 0.001, ****p < 0.0001; ns: not significant.

To identify potential strategies to target ELF3-driven cellular dysfunction under hyperglycemic conditions, drug prediction was performed using the DrugBank database based on the ELF3-associated co-expression network. This analysis identified candidate drugs predicted to mimic or reverse ELF3 activity. Aldesleukin (recombinant human IL-2) was identified as the top-ranking compound. Its strong negative normalized enrichment score (NES) suggested a potential antagonistic effect on ELF3-associated transcriptional programs (Figure 5A). To investigate the potential direct interaction between IL-2 and ELF3, molecular docking analysis was performed. Docking analysis suggested potential structural compatibility between IL-2 and ELF3 (Figure 5B). Following IL-2 treatment, ELF3 protein expression in high-glucose HCECs was reduced in a dose-dependent manner (Figures 5C,D), and SA-β-gal activity decreased, reflecting suppressed cellular senescence (Figures 5E,F).

**FIGURE 6:**
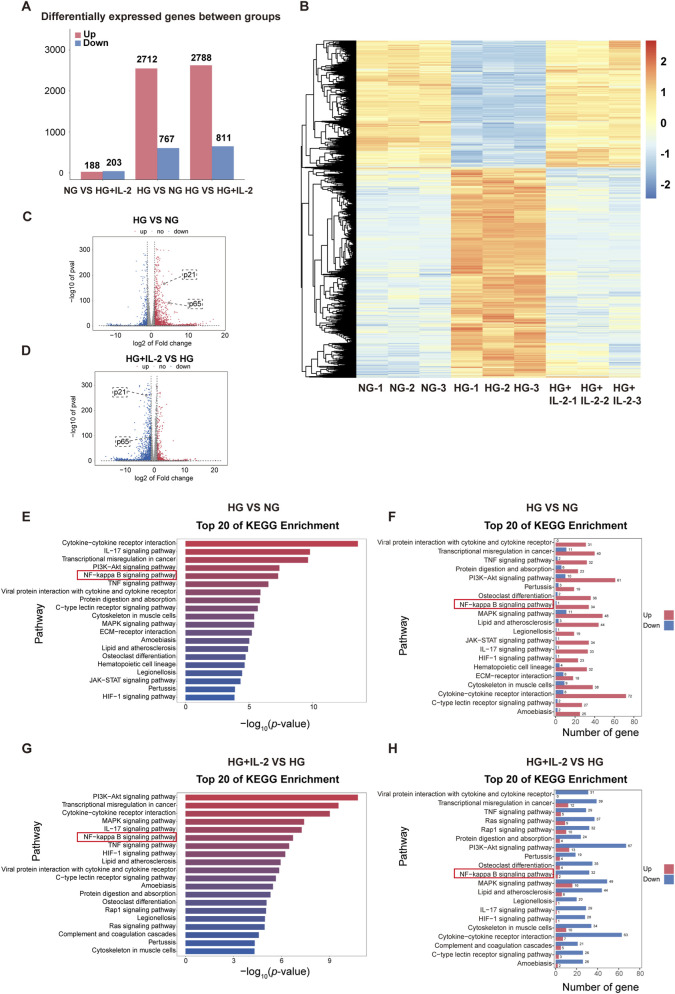
Transcriptomic changes in corneal epithelial cells following IL-2 treatment as revealed by RNA sequencing **(A)** Statistical analysis of differentially expressed genes among experimental groups **(B)** Cluster heatmap analysis of genes among experimental groups **(C,D)** Volcano plot analysis comparing experimental groups **(E,F)** KEGG pathway enrichment analysis in normal-glucose and high-glucose groups **(G,H)** KEGG pathway enrichment analysis in the high-glucose and IL-2-treated groups.

**FIGURE 7:**
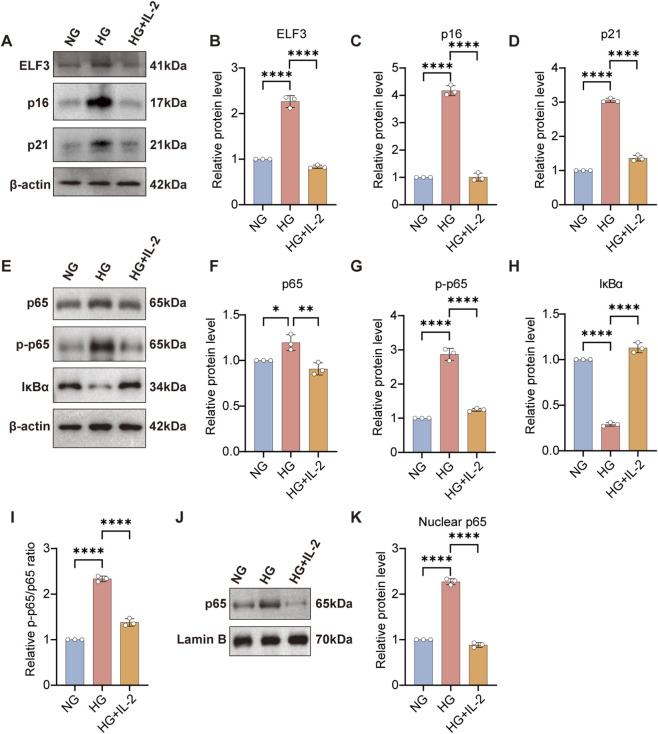
IL-2 suppresses ELF3-associated signaling and attenuates NF-κB pathway activation in HCECs under high-glucose conditions **(A–D)** Western blotting and statistical analysis of ELF3, p16, and p21 protein expression following IL-2 treatment **(E–I)** Western blotting and statistical analysis of p65, p-p65, and IκBα protein expression following IL-2 treatment **(J, K)** Western blotting and statistical analysis of nuclear p65 protein expression. Data are presented as mean ± S.D.; n = 3, *p < 0.05, **p < 0.01, ***p < 0.001, ****p < 0.0001; ns: not significant.

To comprehensively evaluate how IL-2 modulates high-glucose HCECs *via* ELF3, transcriptome sequencing was performed on NG, HG, and HG + IL-2 HCECs. Differential gene expression analysis demonstrated that the HG group contained 2,712 upregulated and 767 downregulated genes compared with the NG group, while it showed 2,788 upregulated and 811 downregulated genes compared with the HG + IL-2 group (Figure 6A). Cluster heatmap analysis demonstrated distinct expression profiles among the three groups, with stable reproducibility across three samples per experimental group (Figure 6B). Volcano plots revealed that p65 and p21 levels were significantly upregulated in the HG group compared with the NG group, whereas the HG + IL-2 group showed downregulation of p65 and p21 relative to the HG group (Figures 6C,D). To identify the key signaling pathways driving high-glucose-induced transcriptional changes, we performed Kyoto Encyclopedia of Genes and Genomes (KEGG) pathway enrichment analysis on differentially expressed genes from the HG *versus* NG comparison. The top 20 enriched pathways (Figures 6E,F) revealed strong enrichment of the NF-κB signaling pathway. We then applied the same analysis to differentially expressed genes from the HG + IL-2 *versus* HG comparison. IL-2 treatment markedly altered the overall pathway landscape (Figures 6G,H), although the NF-κB pathway remained prominently enriched. Additionally, ELF3, p16, p21, p65, and phospho-p65 (p-p65) were significantly upregulated in the HG group (Figures 7A–G), with expression levels decreasing after IL-2 treatment. Conversely, IκBα levels were significantly reduced in the HG group but increased following IL-2 treatment (Figure 7H). The p-p65/p65 ratio in the HG group was upregulated and subsequently decreased after IL-2 intervention (Figure 7I). High glucose also significantly increased p65 expression in HCEC nuclei, which was attenuated by IL-2 treatment (Figures 7J,K). These results demonstrate that high glucose induces IκBα degradation, enhances p65 phosphorylation, and promotes p65 nuclear translocation, reflecting NF-κB pathway activation. ELF3-associated signaling was accompanied by NF-κB pathway activation, while IL-2 attenuated ELF3-associated signaling and NF-κB pathway activation under high-glucose conditions.

### 3.5 ELF3 promotes corneal epithelial senescence and impairs wound healing in diabetic mice, rescued by IL-2 treatment

**FIGURE 8:**
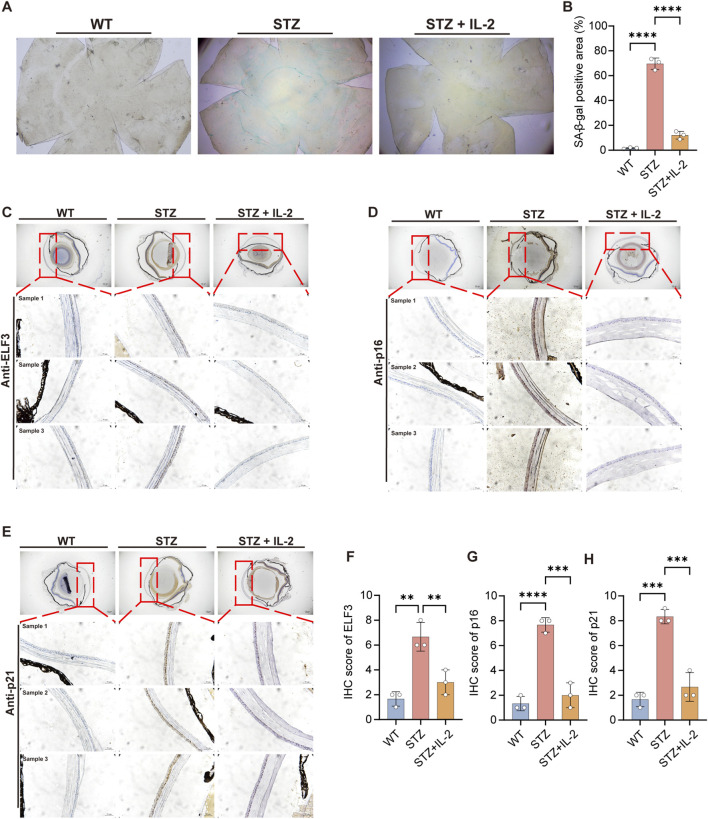
Validation in diabetic mice that IL-2 attenuates corneal epithelial senescence while reducing ELF3 expression **(A,B)** SA-β-gal staining and statistical analysis of the cornea in diabetic mice following IL-2 treatment **(C,F)** Immunohistochemical detection and statistical analysis of ELF3 expression in the corneal epithelium of diabetic mice following IL-2 treatment **(D,G)** Immunohistochemical detection and statistical analysis of p16 expression in the corneal epithelium of diabetic mice following IL-2 treatment **(E,H)** Immunohistochemical detection and statistical analysis of p21 expression in the corneal epithelium of diabetic mice following IL-2 treatment. Scale bar, 100 μm. Data are presented as mean ± S.D.; n = 3, *p < 0.05, **p < 0.01, ***p < 0.001, ns: not significant. Abbreviations: WT, wild-type control mice; STZ, streptozotocin-induced diabetic mice.

**FIGURE 9:**
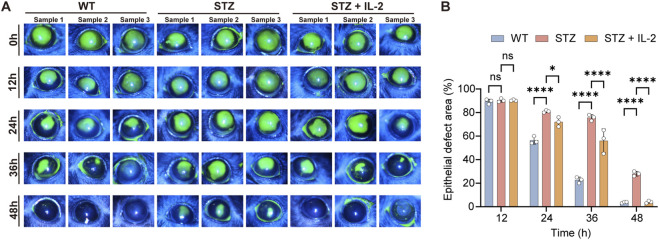
Validation in diabetic mice that IL-2 promotes corneal wound healing under diabetic conditions **(A)** Representative images of sodium fluorescein staining of the corneal wound at different follow-up time points **(B)** Statistical analysis of the corneal wound healing rate. Data are presented as mean ± S.D.; n = 3, *p < 0.05, **p < 0.01, ***p < 0.001, ****p < 0.0001; ns: not significant.

**FIGURE 10:**
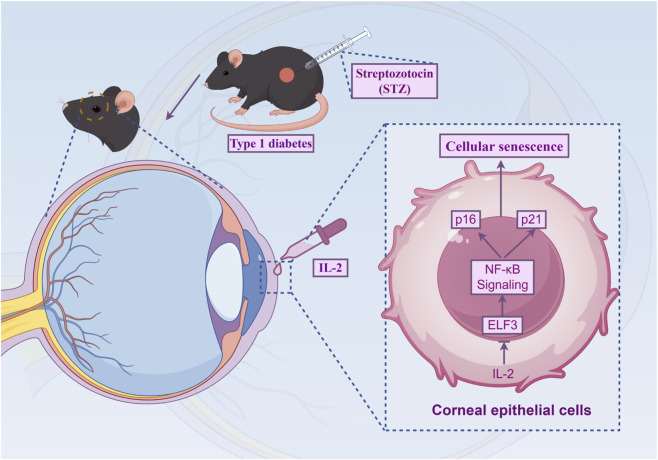
Schematic diagram illustrating ELF3-associated epithelial stress signaling and the modulatory effects of IL-2 on NF-κB pathway activation and corneal senescence under diabetic conditions.

A type 1 diabetes mouse model was established by intraperitoneal STZ injection to explore the function of ELF3 in the diabetic corneal epithelium. Senescence staining indicated that the diabetic corneal epithelium exhibited an increased blue-stained area compared with that of wild-type (WT) control mice, which was markedly reduced after IL-2 treatment (Figures 8A,B). Immunohistochemistry demonstrated that ELF3 levels were significantly elevated in the corneal epithelium of diabetic mice, while IL-2 treatment reversed this upregulation (Figures 8C,F). Similarly, expression changes in p16 and p21 followed this trend (Figures 8D,E,G,H). A corneal epithelial debridement model revealed impaired wound healing in diabetic mice. Administration of IL-2 markedly enhanced the rate of epithelial closure (Figures 9A,B). Collectively, *in vivo* findings confirm that hyperglycemia leads to increased ELF3 expression in the corneal epithelium, driving senescence and delaying wound healing. IL-2 treatment was associated with reduced ELF3 expression, attenuated senescence, and improved epithelial repair. Based on cellular evidence, diabetes-driven corneal senescence is mediated by elevated ELF3, while IL-2 may attenuate senescence and accelerate wound healing through modulation of ELF3-associated signaling and suppression of NF-κB pathway activation (Figure 10).

## 4 Discussion

Numerous investigations have established that diabetes induces premature senescence across multiple tissues, including the cardiovascular system, kidneys, retina, and skeletal muscle (Ali et al., 2024; Espino-Gonzalez et al., 2024; Xu et al., 2024; Gandhi et al., 2025). In the most recognized ocular complications of diabetes—namely diabetic retinopathy—hyperglycemia induces cellular senescence in retinal endothelial and pigment epithelial cells *via* p53/p21 activation, SIRT1 inhibition, Akt dephosphorylation, thereby promoting inflammatory processes and vascular dysfunction (Zhou et al., 2022; Cheng et al., 2024; Hou et al., 2024). Consistent with these reports, the current findings demonstrate that hyperglycemia drives corneal epithelial senescence both *in vitro and in vivo*, and the resulting senescent state impairs corneal wound healing.

Mechanistically, integrative transcriptomic analysis identified ELF3 as a key transcriptional regulator linking hyperglycemia to corneal epithelial senescence. ELF3 is enriched in epithelial tissues and is typically associated with epithelial differentiation under physiological conditions (Yoshida et al., 2000; Lu et al., 2025). However, our data suggest that under hyperglycemic stress, ELF3 functions as a stress-responsive transcription factor associated with the NF-κB signaling pathway and redirects its physiological role toward pathological signaling. This is consistent with previous reports demonstrating that ELF3 is upregulated and mediates pathogenic signaling in human umbilical vascular endothelial cells and podocytes under high-glucose conditions (Sakurai et al., 2019; Wang et al., 2020). Notably, ELF3 has also been shown to drive senescence-associated transcription *via* IRF8-dependent senescence-associated secretory phenotype (SASP) programs (Zhou et al., 2024), further supporting its role as a central mediator of cellular senescence in this study.

From a therapeutic perspective, IL-2 was identified as a potential modulator of ELF3-driven senescence through transcriptome-guided drug prediction and molecular docking. IL-2 is a canonical immunoregulatory cytokine that primarily acts on immune cells. It is clinically approved for oncology indications (Rosenberg, 2014) and has demonstrated immunoregulatory therapeutic benefits in type 1 and type 2 diabetic mice (Nagy et al., 2021; Huo et al., 2025). Corneal epithelial cells also express functional IL-2 receptors (Abu-Romman et al., 2023), and IL-2 signaling is implicated in epithelial proliferation, wound repair, immune regulation, and neuroepithelial integrity (Stepp et al., 2018; Wu et al., 2022). In this study, IL-2 suppressed ELF3 expression, attenuated epithelial senescence and promoted corneal wound healing, raising the possibility that IL-2 exerts epithelial cell-autonomous effects beyond its classical immunomodulatory functions (Marty et al., 2025). The study also found that IL-2 suppresses NF-κB pathway activation in HCECs, which contrasts with the well-established role of IL-2 in activating NF-κB in immune cells (Gomez et al., 1997; Zhou and Meadows, 2003), and may suggest a cell-type- and context-dependent inhibition of NF-κB signaling in HCECs under hyperglycemic stress.

Molecular docking indicated potential structural compatibility between IL-2 and ELF3, raising the possibility that IL-2 may directly interact with ELF3 to modulate its activity or stability. However, whether this potential interaction reflects a direct physical association remains to be experimentally validated. In addition, cytokine-mediated signaling has been reported to influence downstream protein stability and turnover through ubiquitin-proteasome-associated regulatory pathways (Malarde et al., 2009). Therefore, it remains possible that IL-2-mediated signaling contributes to reduced ELF3 protein abundance through indirect regulation of protein stability or transcriptional programs. Together, elucidating the precise molecular mechanism will be an important focus of future study.

The NF-κB pathway is recognized as a master regulator of inflammatory responses and cellular senescence (Zhang et al., 2019; Fu et al., 2025). In this study, RNA sequencing analysis and Western blotting revealed that the suppression of ELF3 by IL-2 was concurrently associated with a marked attenuation of the NF-κB signaling pathway. This coordinated downregulation implies that ELF3-associated signaling was accompanied by NF-κB pathway activation in HCECs, aligning with previous reports that ELF3 modulates NF-κB across various tissues (Li et al., 2015; Feng et al., 2016; He et al., 2021). Although our current work has not yet confirmed the direct regulatory relationship between ELF3 and NF-κB, these findings nominate the ELF3/NF-κB axis as a prospective signaling pathway in diabetic keratopathy, offering a novel perspective on its pathological mechanism. Subsequent investigations are necessary to elucidate the direct molecular interaction between these two factors.

Clinically, these findings suggest several translational implications for the management of diabetic keratopathy. ELF3 may emerge as a promising biomarker for auxiliary diagnosis and severity evaluation, while the topical ocular application of IL-2 serves as a potential therapeutic strategy to alleviate corneal impairment. However, this study has limitations. The diabetic mouse model induced by STZ does not fully recapitulate the metabolic and temporal complexity of corneal disease in humans with diabetes (Furman, 2021). Additionally, transcriptome-guided drug prediction and molecular docking analyses are hypothesis-generating approaches and do not independently establish direct molecular interactions under physiological conditions. The precise molecular basis of IL-2-mediated ELF3 suppression warrants further investigation. Furthermore, the upstream pathways driving ELF3 activation under high-glucose conditions remain to be explored.

In summary, through integrative bioinformatic analysis and experimental validation, this study identifies ELF3 as a central transcriptional mediator linking hyperglycemia to corneal epithelial senescence and demonstrates that IL-2 suppresses ELF3-associated signaling and NF-κB pathway activation while attenuating senescence and promoting corneal wound healing. These findings provide mechanistic insight and therapeutic direction for this clinically challenging complication.

## 5 Conclusion

This research demonstrates that the transcription factor ELF3 drives the senescence of the corneal epithelium in the context of diabetes, and its function is linked to the NF-κB pathway. Additionally, the study establishes that IL-2 functions as a potential modulator of ELF3-associated signaling, providing a potential therapeutic strategy for alleviating cellular senescence and promoting wound healing in diabetic keratopathy.

## Data Availability

The two publicly available sequencing datasets can be found in the NCBI GEO databases. The original RNA-Seq data generated for this study have been uploaded in the National Genomics Data Center database under accession number HRA016841.
