# Supplementary material for: ELF3 promotes hyperglycemia-induced corneal epithelial senescence and IL-2 attenuates ELF3-associated signaling
Source: Front Mol Biosci. 2026 Jun 24;13:1812782. doi: 10.3389/fmolb.2026.1812782 (PMC13341422; doi:10.3389/fmolb.2026.1812782)
Supplement: Supplementary file 1 [file Supplementaryfile1.doc]

Supplementary Material

# Supplementary Figures


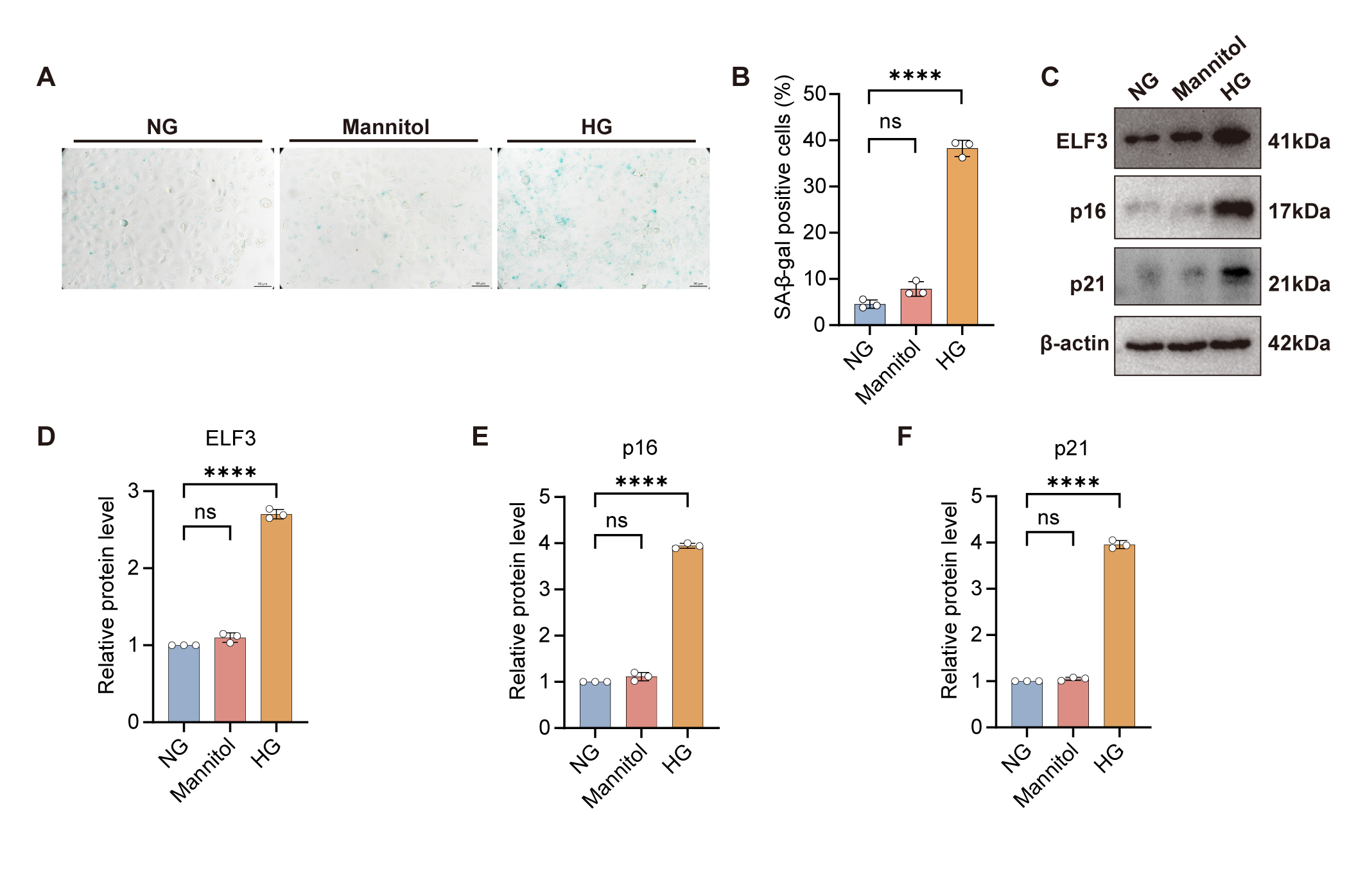


**Supplementary Figure 1.** High glucose induces senescence in HCECs independent of osmotic pressure (mannitol control). **(A, B)** SA-β-gal staining and statistical analysis of HCECs under normal glucose (NG), high glucose (HG, 35 mM), and mannitol conditions. **(C–F)** Western blotting and statistical analysis of ELF3, p16, and p21 protein expression in HCECs under NG, HG, and mannitol conditions. Data are presented as mean ± S.D.; n=3, **p* < 0.05, ‌***p* < 0.01, ****p* < 0.001, *****p* < 0.0001; ns: not significant.
